# Supplementary material for: Cortical-limbic circuit dynamics of approach-avoidance conflict in humans
Source: Nat Commun. 2026 Mar 12;17:3867. doi: 10.1038/s41467-026-70287-5 (PMC13125241; doi:10.1038/s41467-026-70287-5)
Supplement: Supplementary file 1 — Supplementary Information [file 41467_2026_70287_MOESM1_ESM.pdf]

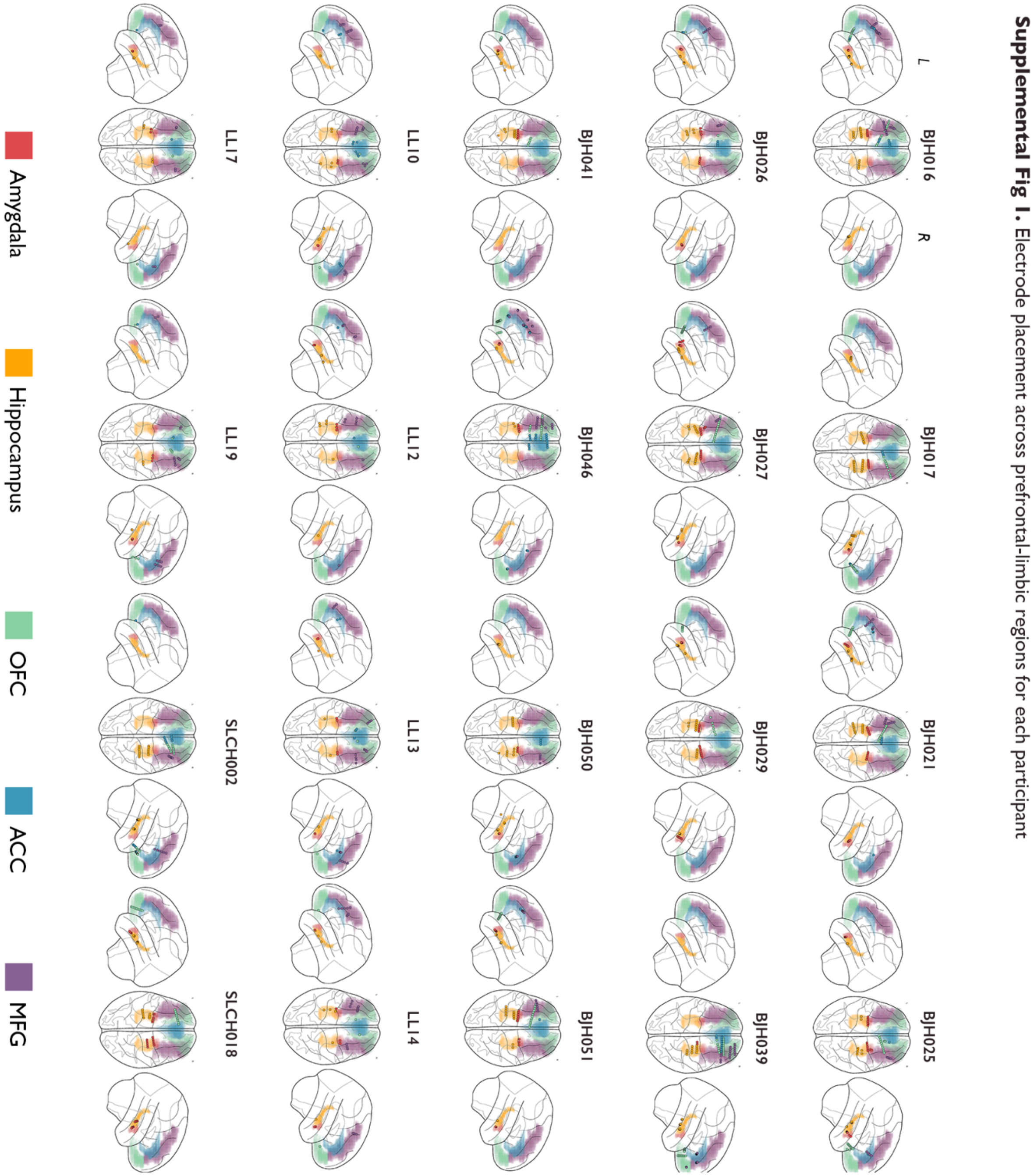

Supp Fig. 1. Electrode placement for each of the twenty patients. Electrodes were placed across the prefrontal and limbic regions. Colored shading indicates region and dots indicate electrode.

Supplemental Fig 2. Time-frequency representations of conflict-free trials, time-locked to avoidance choice in all regions

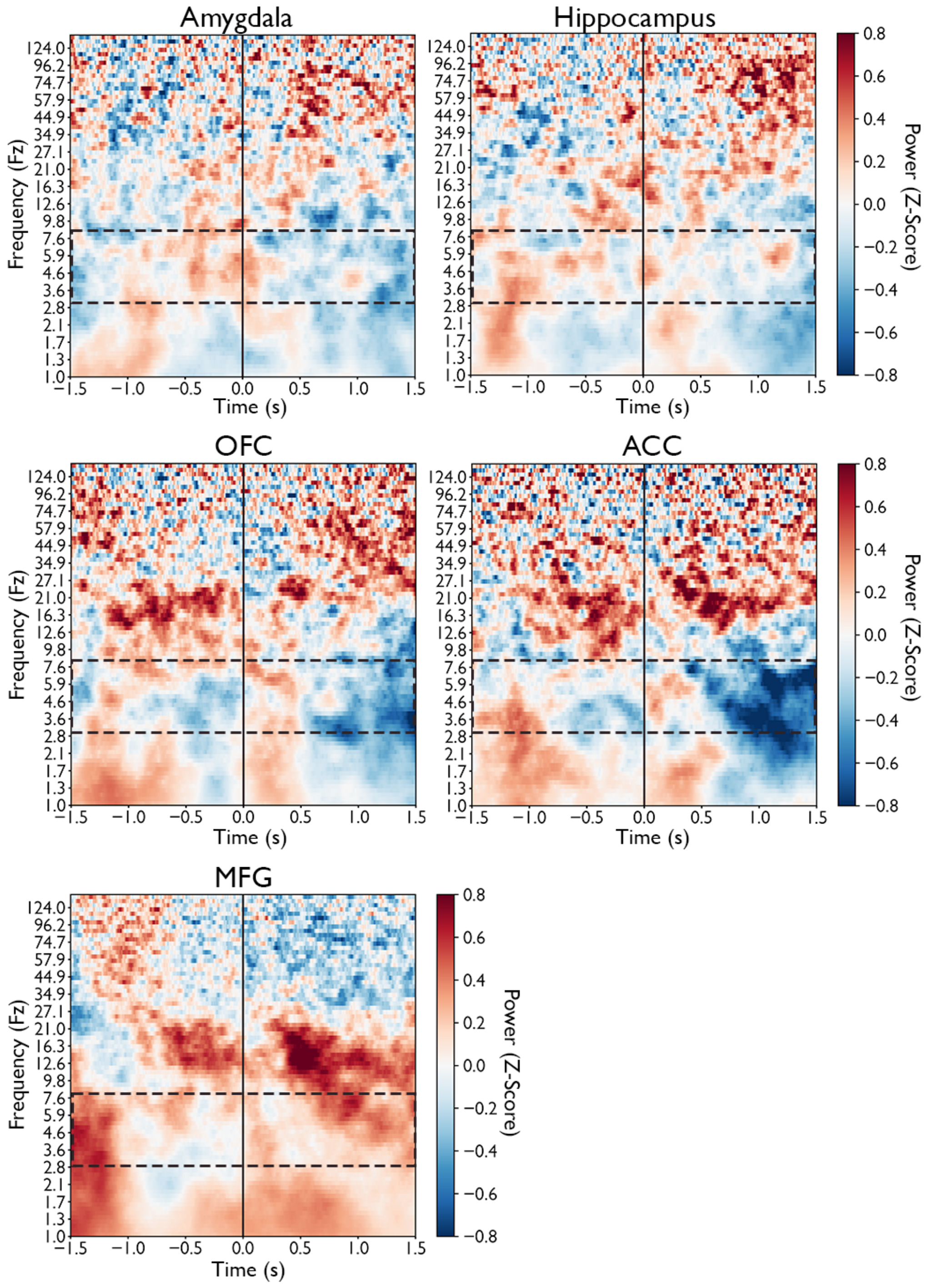

Supp Fig. 2. Time-frequency analysis of neural activity time-locked to the final choice to stop approaching and begin avoiding in conflict-free (reward-only, no ghost) trials across all electrodes in the limbic regions (hippocampus, amygdala, OFC and ACC) and the MFG. Red (blue) indicates increases (decreases) in z-scored power. Dotted box indicates the theta band (3-8Hz).

Supplemental Table 1

**Subject**	**Age (Years)**	**Sex**	**Amygdala**	**Hippocampus**	**OFC**	**ACC**	**MFG**
SUB016	24	M	4	15	11	7	12
SUB017	30	M	5	20	10	3	0
SUB021	38	F	10	11	10	4	9
SUB025	45	F	12	8	12	1	4
SUB026	40	M	7	5	1	3	4
SUB027	37	M	12	16	14	0	4
SUB029	34	F	11	13	6	0	0
SUB039	65	F	2	11	16	10	16
SUB041	42	M	5	7	5	0	0
SUB046	60	F	4	2	16	16	13
SUB050	28	F	3	12	0	4	4
SUB051	41	F	9	11	12	1	6
SUB010	33	F	2	5	1	6	10
SUB012	33	M	3	5	1	1	6
SUB013	40	M	4	2	2	3	11
SUB014	32	M	3	4	2	0	11
SUB015	54	M	2	4	1	1	4
SUB019	30	F	3	2	4	1	9
SUB0002	21	M	0	13	11	8	6
SUB018	18	F	14	8	9	0	0

Supp. Table 1: Demographic and electrode information for the intracranial sample. Age, self-reported sex, and electrode count for each region are included.

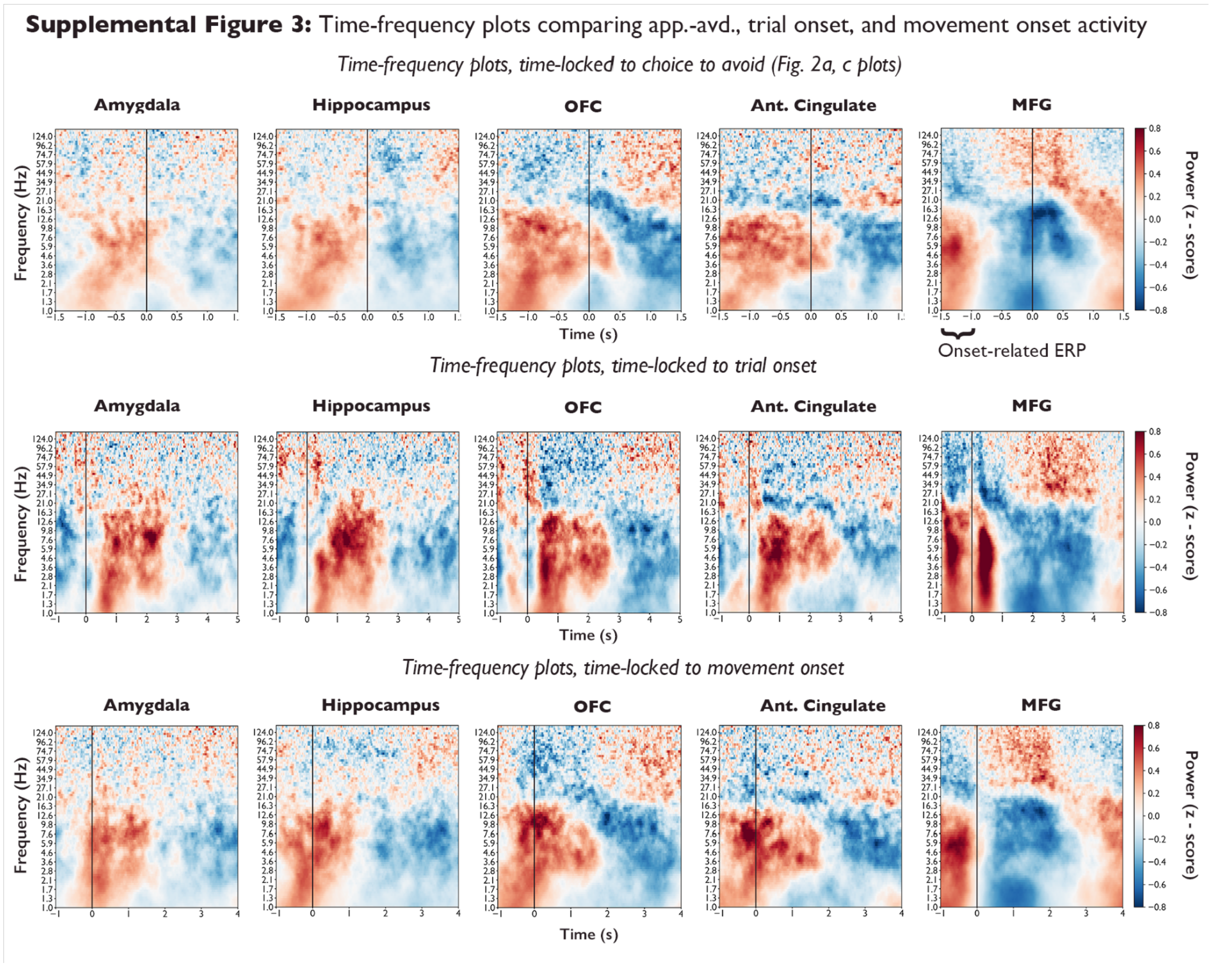

Supp Figure 3: Time-frequency plots of neural activity in each region in conflict trials time-locked to three different trial events: (top) the final choice to stop approaching and begin avoiding, (middle) trial onset, and (bottom) movement onset. Red (blue) indicates increases (decreases) in power. Power was log-transformed and z-scored to the mean power across the time window. Though it appears as though the MFG has increased theta power at the beginning of the approach period while time-locking to the decision to avoid, this is likely nonoscillatory, event-related activity, rather than an increase in theta oscillations.

To account for trial-onset related activity, which is unlikely to reflect putative oscillatory activity, we excluded timepoints after the trial began but before movement onset. Movement onset in the trial begins on average 1.02+/- 0.46 seconds after trial onset, across patients. In the TFRs above, this tended to remove the portion of the transient changes in power, while keeping the changes that endured during the approach period. While nonoscillatory changes can be interesting in of themselves, our hypotheses centered on the theta band, which led us to exclude activity likely not related to oscillations.

Supp. Fig. 4: Theta Oscillations in the Middle Frontal Gyrus

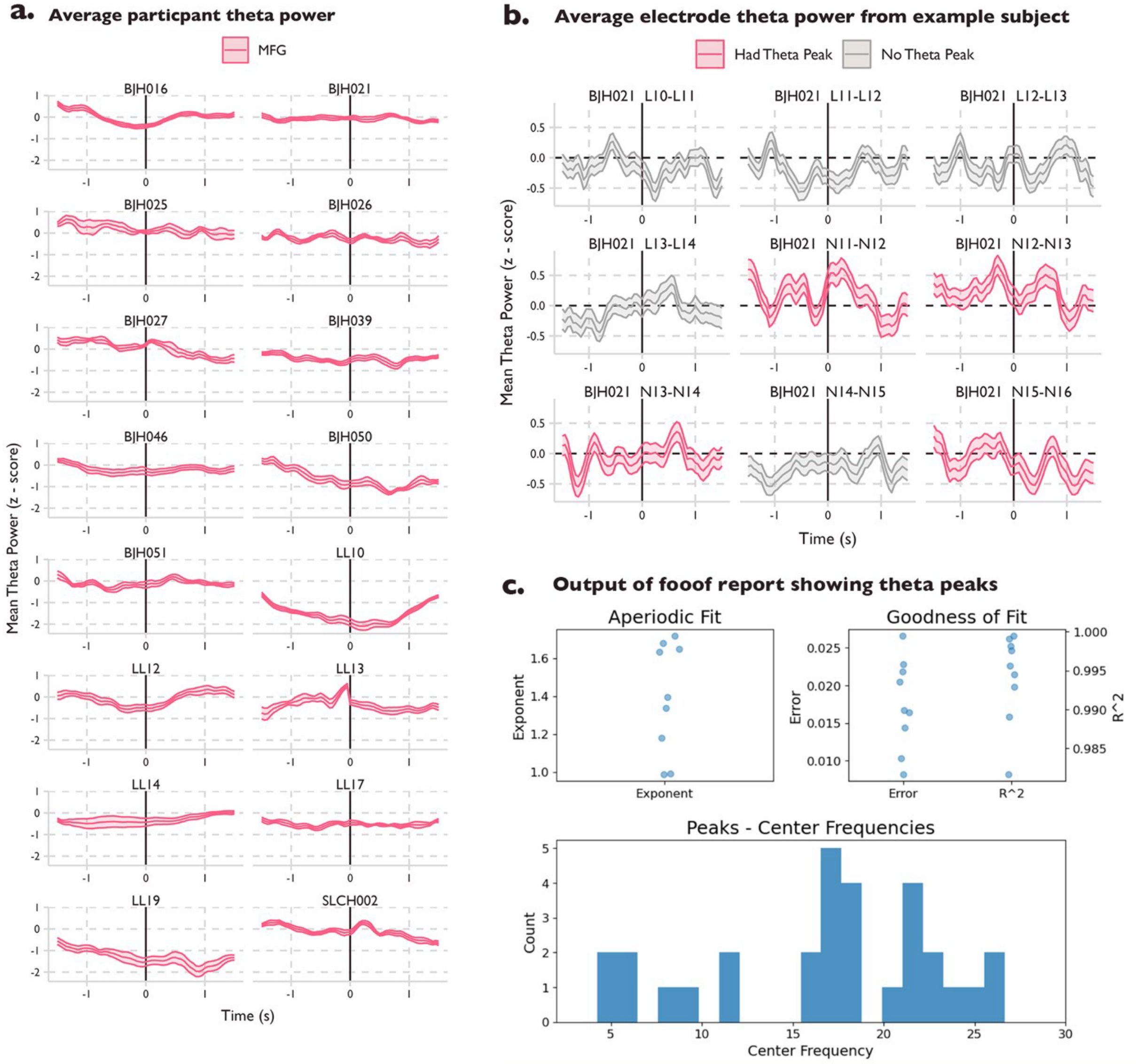

Supp Fig. 4. **a** Time-course of theta power in the MFG for each patient across approach and avoidance windows. 0 indicates the time the patient stopped approaching and began avoiding. Shading is the standard error of the mean power across subjects. **b** Average time-course of theta power by electrode for an example subject. While this example subject had an average near 0 of z-scored theta power, when looking at the electrode level there are dynamics in the time-course. 0 indicates the time the patient stopped approaching and began avoiding. Shading is the standard error of the mean and the color indicates if that electrode had a peak in the theta band, as assessed by the FOOOF (Fitting Oscillations and One-Over-F) algorithm—which decomposes the power spectrum into its aperiodic (1/f) and periodic components. **c** Output for a group-level FOOOF report run on all the electrodes for the example participant in panel b. Output shows high R-squared values and low error. While peaks in the beta-band were more common, 4/12 electrodes also showed peaks in the theta-band.

Supp Table 2. Percentage of electrode pairs with significantly elevated theta coherence for each region.

**Mean Percentage of Significant Electrode Pairs**				
	Mean	SD	Min	Max
Hippocampus				
Imaginary Coherence	36%	16%	10%	68%
Pairwise Phase Consistency	46%	22%	15%	88%
Phase Lag Index	37%	17%	6%	67%
Amygdala				
Imaginary Coherence	36%	12%	17%	62%
Pairwise Phase Consistency	52%	19%	17%	83%
Phase Lag Index	38%	14%	17%	67%
OFC				
Imaginary Coherence	38%	17%	8%	85%
Pairwise Phase Consistency	50%	18%	17%	94%
Phase Lag Index	40%	17%	13%	88%
ACC				
Imaginary Coherence	45%	16%	20%	75%
Pairwise Phase Consistency	59%	17%	21%	83%
Phase Lag Index	46%	18%	11%	83%
MFG				
Imaginary Coherence	33%	18%	14%	74%
Pairwise Phase Consistency	44%	21%	14%	90%
Phase Lag Index	34%	18%	14%	76%

Supp. Table 2. Table showing the percentages of significant electrode pairs in each of the 5 regions. The mean, standard deviation (SD), min and max percentage across patients and within regions are shown for each of the three connectivity metrics.

Supp Fig 5. Prefrontal-limbic regions cohere in theta during approach

a-b Prefrontal and subcortical regions form subnetworks within a wider theta circuit

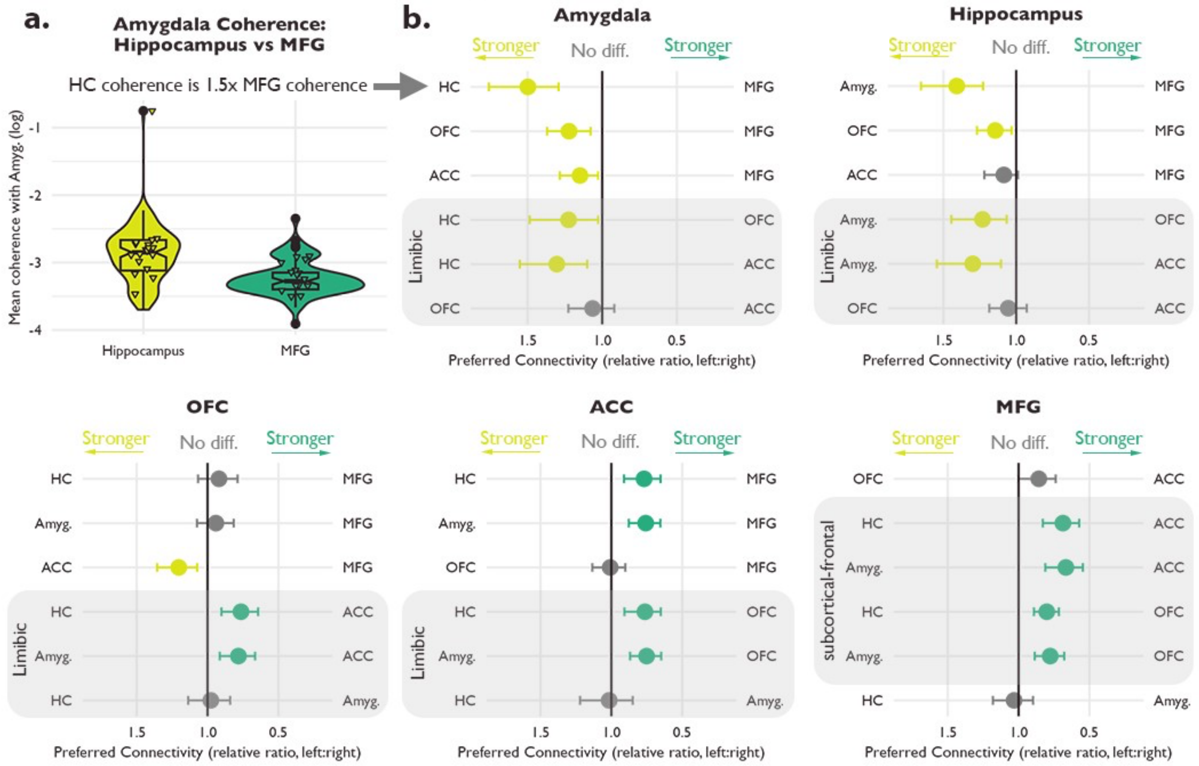

Supp Fig. 5 . Full interregional comparison of theta-band imaginary coherence strength. Theta coherence in each region was modeled with all other regions as predictors, similar to a frequentist ANOVA. **a** Comparison of estimated theta coherence strength between the Hippocampus and MFG, within the Amygdala. X-axis is the region, y-axis if the log-scaled estimated coherence with the amygdala. This plot is summarized in the first line of the first subplot in b. Box plots depict median and interquartile range across electrodes-pairs, with whiskers covering most extreme values except outliers. **b** Each row on the y-axis compares the coherence strength between the left and right regions. Dots indicate the estimated relative ratio of preferred connectivity. Lines indicate the 95% credible interval. Grey indicates the credible interval includes 1, meaning there is no difference in coherence strength between the two regions. For the limbic subplots, grey shading highlights within limbic comparisons, while for the MFG, the grey shading highlights subcortical-frontal comparisons.

Supp Fig 6. Similar subnetworks using alternate coherence metrics

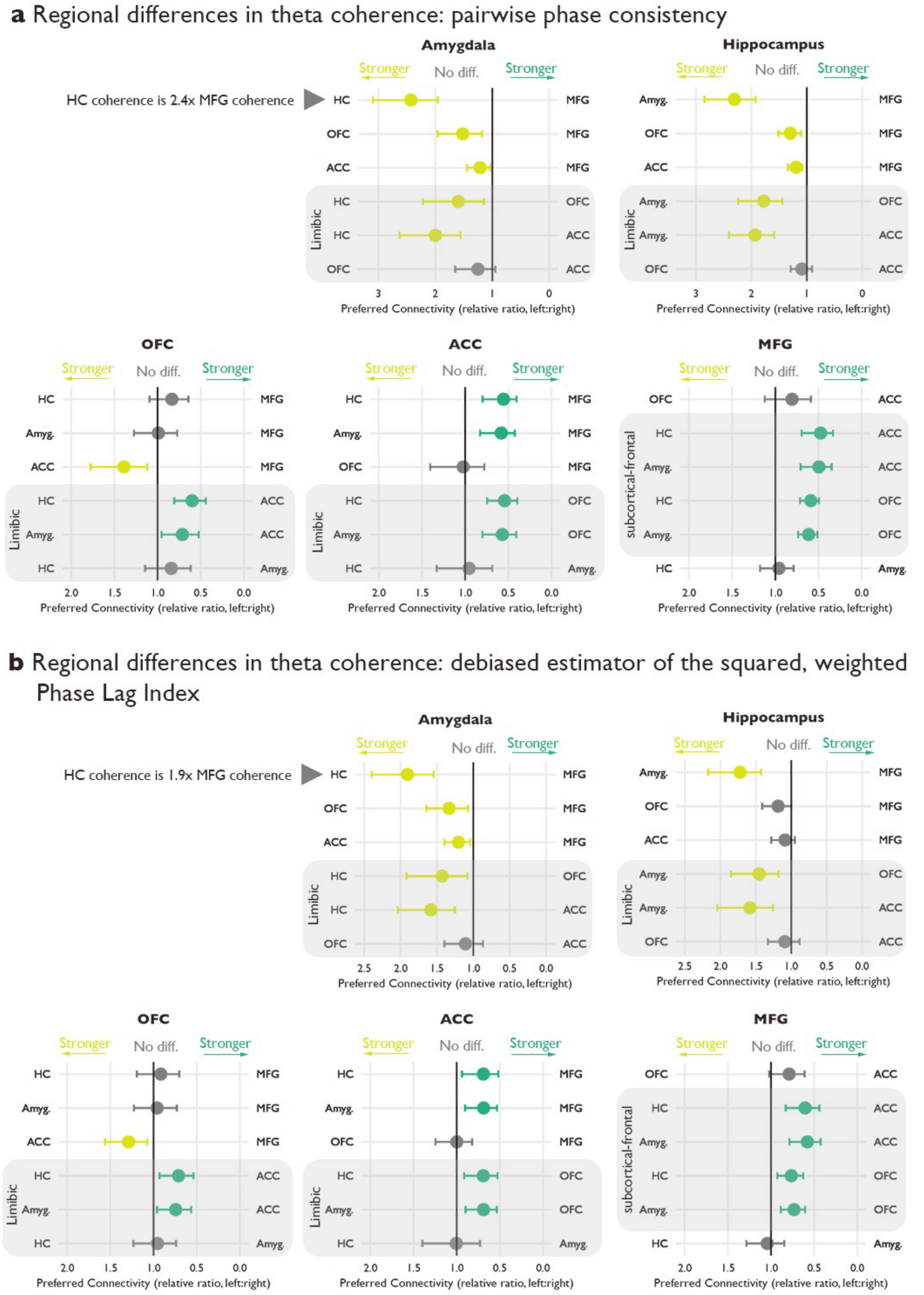

Supp Fig. 6. Theta connectivity profiles using **a** pairwise phase consistency **b** phase lag index. Theta coherence in each region was modeled with all other regions as predictors. Each row on the y-axis compares the coherence strength between the left and right regions. Dots indicate the estimated relative ratio of preferred connectivity. Lines indicate the 95% credible interval. Grey indicates the credible interval includes 1, meaning there is no difference in coherence strength between the two regions. For the limbic subplots, grey shading highlights within limbic comparisons, while for the MFG, the grey shading highlights subcortical-frontal comparisons.

Supp Table 3. Theta connectivity strength ratios and their 95% confidence intervals, estimated error, and convergence metrics across the three connectivity metrics

Region	Comparison	Metric	Mean	l-95% CI	u-95% CI	Est.Error	Rhat	Bulk_ESS
Amygdala Coherence	Hippocampus Ant.Cingulate	PPC	2	1.56	2.63	0.13	1	6210.77
		PLI	1.58	1.25	2.04	0.12	1	7952.22
		IMCOH	1.3	1.1	1.55	0.09	1	8677.27
	Hippocampus - OFC	PPC	1.6	1.15	2.22	0.17	1	5112.92
		PLI	1.43	1.08	1.92	0.14	1	6466.17
		IMCOH	1.23	1.03	1.49	0.09	1	7495.55
	Hippocampus - MFG	PPC	2.43	1.96	3.1	0.12	1	5590.09
		PLI	1.91	1.55	2.4	0.11	1	6942.64
		IMCOH	1.5	1.29	1.76	0.08	1	7582.33
	Ant.Cingulate - OFC	PPC	0.8	0.61	1.05	0.14	1	6037.59
		PLI	0.9	0.72	1.15	0.12	1	7236.54
		IMCOH	0.94	0.82	1.09	0.07	1	8807.66
	Ant.Cingulate - MFG	PPC	1.21	1.04	1.44	0.08	1	8890.59
		PLI	1.2	1.04	1.4	0.07	1	13269.23
		IMCOH	1.15	1.03	1.28	0.06	1	11399.59
	OFC - MFG	PPC	1.52	1.18	1.96	0.13	1	4942.21
		PLI	1.33	1.07	1.64	0.11	1	6436.95
		IMCOH	1.22	1.08	1.37	0.06	1	6426.08
Hippocampus Coherence	Ant.Cingulate - OFC	PPC	0.92	0.77	1.1	0.09	1	4154.52
		PLI	0.71	0.54	0.88	0.09	1	4627.79
		IMCOH	0.95	0.84	1.08	0.06	1	7121.59
	Ant.Cingulate - Amygdala	PPC	0.52	0.42	0.63	0.11	1	5411.76
		PLI	0.63	0.49	0.79	0.12	1	5306.36
		IMCOH	0.77	0.65	0.91	0.08	1	6767.99
	Ant.Cingulate - MFG	PPC	1.19	1.08	1.34	0.05	1	7026.03
		PLI	1.09	0.95	1.28	0.08	1	5902.45
		IMCOH	1.08	0.99	1.22	0.05	1	6800.81
	OFC - Amygdala	PPC	0.56	0.45	0.69	0.11	1	5318.97
		PLI	0.69	0.54	0.85	0.11	1	6212.42
		IMCOH	0.81	0.69	0.95	0.07	1	8111.63
	OFC - MFG	PPC	1.3	1.1	1.51	0.08	1	3676.91
		PLI	1.19	1	1.41	0.09	1	4689.38
		IMCOH	1.14	1.03	1.27	0.05	1	5719.22

	Amygdala - MFG	PPC	2.31	1.92	2.85	0.1	1	5016.48
		PLI	1.72	1.42	2.07	0.11	1	4595.4
		IMCOH	1.41	1.23	1.65	0.07	1	5789.61
OFC Coherence	Hippocampus Ant.Cingulate	PPC	0.6	0.44	0.81	0.1	1	5702.87
		PLI	0.69	0.53	0.91	0.1	1	5537.6
		IMCOH	0.76	0.64	0.9	0.09	1	5733.13
	Hippocampus - Amygdala	PPC	0.84	0.62	1.14	0.16	1	4270.48
		PLI	1.09	0.97	1.23	0.13	1	5380.69
		IMCOH	0.98	0.84	1.14	0.08	1	7321.22
	Hippocampus - MFG	PPC	0.83	0.64	1.09	0.13	1	4204.27
		PLI	0.92	0.79	1.07	0.08	1	3334.17
		IMCOH	0.92	0.79	1.07	0.08	1	3571.11
	Ant.Cingulate - Amygdala	PPC	1.4	1.05	1.91	0.2	1	5596.56
		PLI	1.34	1.04	1.77	0.17	1	4393.3
		IMCOH	1.09	0.91	1.28	0.1	1	4920.29
	Ant.Cingulate - MFG	PPC	1.39	1.12	1.77	0.17	1	4628.01
		PLI	1.56	1.09	1.56	0.09	1	4753.71
		IMCOH	1.2	1.03	1.36	0.06	1	5742.93
	Amygdala - MFG	PPC	1.27	1.03	1.56	0.07	1	3307.7
		PLI	1.23	1	1.53	0.09	1	3119.64
		IMCOH	1.07	0.92	1.25	0.07	1	2649.24
ACC Coherence	Hippocampus - OFC	PPC	0.54	0.4	0.74	0.16	1	5344.18
		PLI	0.69	0.53	0.91	0.11	1	6299.5
		IMCOH	0.77	0.65	0.91	0.08	1	5089.88
	Hippocampus - Amygdala	PPC	0.95	0.69	1.33	0.17	1	7274.48
		PLI	1.04	0.85	1.29	0.11	1	7591.34
		IMCOH	1.02	0.85	1.22	0.09	1	5739.08
	Hippocampus - MFG	PPC	0.56	0.4	0.8	0.17	1	3424.54
		PLI	0.69	0.52	0.94	0.15	1	4115.56
		IMCOH	0.77	0.65	0.91	0.09	1	3670.91
	OFC - Amygdala	PPC	1.75	1.25	2.44	0.17	1	7180.89
		PLI	1.45	1.11	1.87	0.13	1	7740.18
		IMCOH	1.33	1.15	1.54	0.07	1	7833.11
	OFC - MFG	PPC	1.02	0.78	1.4	0.15	1	2893.81
		PLI	1	0.82	1.25	0.11	1	4044.41
		IMCOH	1.01	0.9	1.13	0.06	1	4296.64

	Amygdala - MFG	PPC	0.58	0.43	0.83	0.17	1	4037.23
		PLI	0.69	0.53	0.9	0.13	1	5225.58
		IMCOH	0.76	0.65	0.88	0.07	1	4476.46
MFG Coherence	Hippocampus Ant.Cingulate	PPC	0.48	0.33	0.69	0.18	1	4195.15
		PLI	0.66	0.44	0.83	0.16	1	5394.4
		IMCOH	0.69	0.57	0.83	0.09	1	7892.21
	Hippocampus - Amygdala	PPC	0.96	0.79	1.18	0.1	1	6659.15
		PLI	1.04	0.85	1.29	0.11	1	8904.23
		IMCOH	1.03	0.9	1.18	0.07	1	10835.17
	Hippocampus - OFC	PPC	0.59	0.49	0.71	0.09	1	3791.46
		PLI	0.76	0.63	0.93	0.1	1	4617.98
		IMCOH	0.8	0.72	0.89	0.06	1	7890.27
	Ant.Cingulate - Amygdala	PPC	2.02	1.41	2.87	0.18	1	3489.54
		PLI	1.73	1.27	2.35	0.16	1	5950.72
		IMCOH	1.5	1.23	1.83	0.1	1	8560.84
	Ant.Cingulate - OFC	PPC	1.24	0.89	1.7	0.16	1	3603.86
		PLI	1.27	0.98	1.63	0.13	1	5928.85
		IMCOH	1.2	1	1.35	0.08	1	7590.22
	Amygdala - OFC	PPC	0.65	0.51	0.74	0.09	1	4508.78
		PLI	0.73	0.6	0.89	0.07	1	6344.24
		IMCOH	0.78	0.68	0.89	0.07	1	8728.89

Supp. Table 3. Connectivity strength ratios and their corresponding 95% confidence intervals (l-95% CI & u-95% CI), estimated error, and two convergence metrics (Rhat and Bulk Estimated Sample Size) for each region contrast, within each region, for all three connectivity metrics. These results come from a Bayesian mixed effects model run within each of the five regions to predict the average theta coherence during the approach window (1500ms preceding the avoidance decision), using a four-level factor representing the remaining regions as the predictor. Connectivity strength ratios comparing each pair of regions within a region were then calculated.

Supplemental Table 4. Posterior Predictive Checks for 'Theta Coherence ~ Region' model

Posterior Predictive Checks: Theta Coherence ~ Region					
	Statistic	Observed Value	Posterior Predictive Mean	Credible Interval	Posterior Predictive P Value
Amygdala	**PPC**				
	Mean	-5.0112562	-5.0109124	[-5.051, -4.964]	0.5
	Standard Deviation	0.9911363	0.9937864	[0.961, 1.025]	0.57
	**PLI**				
	Mean	-3.9392873	-3.9399313	[-3.982, -3.898]	0.48
	Standard Deviation	0.8322597	0.8350218	[0.802, 0.874]	0.57
	**IMCOH**				
	Mean	-3.0220568	-3.0235595	[-3.052, -2.995]	0.52
	Standard Deviation	0.5608367	0.5632271	[0.544, 0.583]	0.61
Hippocampus	**PPC**				
	Mean	-5.0983353	-5.1020627	[-5.125, -5.08]	0.44
	Standard Deviation	0.8094896	0.8105779	[0.786, 0.834]	0.5
	**PLI**				
	Mean	-3.9392681	-3.940503	[-3.966, -3.916]	0.5
	Standard Deviation	0.6875272	0.6884104	[0.669, 0.708]	0.49
	**IMCOH**				
	Mean	-3.0305881	-3.0317367	[-3.054, -3.013]	0.48
	Standard Deviation	0.4844154	0.4853753	[0.467, 0.501]	0.55
OFC	**PPC**				
	Mean	-4.9325216	-4.9319141	[-4.959, -4.912]	0.55
	Standard Deviation	0.8963513	0.8970578	[0.866, 0.921]	0.59
	**PLI**				
	Mean	-3.8465322	-3.8487639	[-3.876, -3.823]	0.44
	Standard Deviation	0.7881697	0.7883251	[0.767, 0.81]	0.49
	**IMCOH**				
	Mean	-2.9533249	-2.9530203	[-2.972, -2.936]	0.52
	Standard Deviation	0.528249	0.5287812	[0.516, 0.541]	0.56

ACC	**PPC**				
	Mean	-4.7461005	-4.7469097	[-4.806, -4.703]	0.51
	Standard Deviation	0.9938289	0.9983173	[0.961, 1.034]	0.6
	**PLI**				
	Mean	-3.6963435	-3.6976578	[-3.747, -3.65]	0.5
	Standard Deviation	0.9024046	0.9077206	[0.868, 0.941]	0.62
	**IMCOH**				
	Mean	-2.8681809	-2.8697937	[-2.902, -2.839]	0.39
	Standard Deviation	0.5755032	0.5780758	[0.56, 0.604]	0.56
MFG	**PPC**				
	Mean	-4.9665244	-4.9686332	[-5.001, -4.937]	0.46
	Standard Deviation	0.9231406	0.9271355	[0.902, 0.952]	0.6
	**PLI**				
	Mean	-3.8542984	-3.8525964	[-3.879, -3.827]	0.57
	Standard Deviation	0.8024406	0.804357	[0.78, 0.831]	0.53
	**IMCOH**				
	Mean	-2.9851885	-2.9847188	[-3.001, -2.963]	0.47
	Standard Deviation	0.5273133	0.527762	[0.511, 0.545]	0.48

Supp. Table 4. Posterior predictive checks on the mean and standard deviation for the models using region to predict theta coherence. The observed value is the observed mean or standard deviation of the data. The mean, 95% confidence intervals, and posterior predictive values (which should be near .5) of the posterior distribution of theta coherence are shown. In all cases, the observed data match the fit posterior distribution.

Supp Table 5. Estimates & posterior predictive checks for models estimating the effect of time on theta coherence, including alternate timepoints & alternate coherence metrics

Effect of Time on Theta Coherence

		Model Estimates							Posterior Predictive Checks							
Metric	Timepoint	Estimate	Est.Error	l-95% CI	u-95% CI	Rhat	Bulk_ESS	Tail_ESS	True Mean	True SD	Posterior Predictive Mean	CI- Mean	Posterior Predictive SD	CI- SD	Post. Pred. Mean p value	Post. Pred. SD p value
**Approach**																
Im. Coh.	*1.5 (s)*	0.06	0.04	-0.01	0.14	1.00	445.27	1332.97	0	1	0.000	[-0.008, 0.008]	1.000	[0.994, 1.008]	0.43	0.53
Im. Coh.	*1.6 (s)*	0.08	0.03	0.01	0.14	1.00	3196.19	5592.32	0	1	0.000	[-0.008, 0.008]	1.000	[0.994, 1.005]	0.50	0.50
Im. Coh.	*1.7 (s)*	0.09	0.03	0.03	0.14	1.00	4131.51	6590.06	0	1	0.000	[-0.007, 0.009]	1.001	[0.993, 1.009]	0.49	0.55
Im. Coh.	*1.8 (s)*	0.09	0.03	0.03	0.14	1.00	4093.91	6303.93	0	1	-0.001	[-0.009, 0.011]	1.000	[0.993, 1.006]	0.40	0.49
Im. Coh.	*2 (s)*	0.09	0.02	0.05	0.14	1.00	7680.42	9704.80	0	1	0.000	[-0.009, 0.007]	1.000	[0.992, 1.006]	0.52	0.58
PPC	*2 (s)*	0.07	0.02	0.03	0.10	1.00	9293.28	8836.05	0	1	-0.001	[-0.008, 0.006]	1.001	[0.995, 1.007]	0.43	0.57
PLI	*2 (s)*	0.08	0.02	0.04	0.13	1.00	10046.50	10629.59	0	1	0.000	[-0.005, 0.006]	1.000	[0.994, 1.004]	0.46	0.50
**Avoid**																
Im. Coh.	*1.5 (s)*	-0.13	0.04	-0.20	-0.05	1.00	5387.01	6839.51	0	1	0.000	[-0.012, 0.009]	1.001	[0.993, 1.008]	0.48	0.56
Im. Coh.	*1.6 (s)*	-0.13	0.03	-0.19	-0.06	1.01	1080.11	2326.39	0	1	0.000	[-0.009, 0.01]	1.000	[0.993, 1.008]	0.55	0.51
Im. Coh.	*1.7 (s)*	-0.12	0.03	-0.18	-0.06	1.00	4770.02	6872.33	0	1	0.000	[-0.009, 0.007]	1.000	[0.993, 1.008]	0.50	0.48
Im. Coh.	*1.8 (s)*	-0.12	0.03	-0.18	-0.06	1.00	5651.34	7151.48	0	1	0.001	[-0.006, 0.008]	1.000	[0.994, 1.007]	0.56	0.42
Im. Coh.	*2 (s)*	-0.11	0.03	-0.16	-0.06	1.00	3520.35	4769.50	0	1	0.000	[-0.009, 0.009]	1.000	[0.993, 1.006]	0.44	0.54
PPC	*2 (s)*	-0.12	0.03	-0.17	-0.07	1.00	7365.40	10049.77	0	1	0.000	[-0.005, 0.006]	1.000	[0.995, 1.005]	0.49	0.51
PLI	*2 (s)*	-0.11	0.03	-0.18	-0.05	1.00	5228.66	8321.24	0	1	0.000	[-0.007, 0.007]	1.000	[0.994, 1.007]	0.52	0.45

Supp. Table 5. Model estimates and posterior predictive checks for the models predicting theta coherence using time in the approach and avoidance windows, across the three connectivity metrics. Models were run using the following timepoints using imaginary coherence: 1.5s, 1.6s, 1.7s, 1.8s, 2s before the decision to avoid. Models were run using pairwise phase consistency (PPC) and weighted and debiased Phase Locking Index (PLI) in the 2 second before the avoidance decision. The true mean and SD are within the 95% confidence intervals, and the posterior predictive values are near 0.5. for all models.

Supplemental Table 6. Full results for a model estimating the effect of approach time on theta synchrony, with interactions for each region pair

	Region Pair	Beta	CI Lower	CI Upper	P+	Significant
Theta Synchrony	*Amyg. ~ ACC*	0.068	0.042	0.094	1	Significant
	Amyg. ~ MFG	0.007	-0.015	0.029	0.739	Not Significant
	HC ~ ACC	0.024	0	0.05	0.973	Not Significant
	HC ~ Amyg.	0.052	0.035	0.07	1	Significant
	HC ~ MFG	0.017	-0.003	0.037	0.952	Not Significant
	MFG ~ ACC	0.033	0.016	0.049	1	Significant
	OFC ~ ACC	0.061	0.044	0.077	1	Significant
	OFC ~ Amyg.	0.048	0.029	0.065	1	Significant
	OFC ~ HC	0.038	0.022	0.055	1	Significant
	OFC ~ MFG	0.054	0.037	0.069	1	Significant
HFA Synchrony	*Amyg. ~ ACC*	-0.014	-0.036	0.008	0.11	Not Significant
	Amyg. ~ MFG	0.015	-0.003	0.034	0.946	Not Significant
	HC ~ ACC	0.01	-0.012	0.032	0.811	Not Significant
	HC ~ Amyg.	0.007	-0.006	0.019	0.863	Not Significant
	HC ~ MFG	-0.019	-0.036	-0.003	0.009	Significant
	MFG ~ ACC	0.005	-0.007	0.015	0.798	Not Significant
	OFC ~ ACC	0.018	0.006	0.029	0.999	Significant
	OFC ~ Amyg.	-0.003	-0.016	0.011	0.35	Not Significant
	OFC ~ HC	0.006	-0.006	0.018	0.855	Not Significant
	OFC ~ MFG	0.012	0.001	0.022	0.982	Significant

Supp. Table 6. Model results showing how approach times correlate with theta and HFA synchrony during the approach window. For each region pair, the beta coefficient, upper and lower 95% confidence intervals, P+ values, and significance are shown.

Supplemental Table 7. Directed connectivity results using both a Net Granger analysis and a cross-correlation analysis

Net Granger and Cross Correlation Results by Region Pair					
Region Pair	P+	Total Electrode Pairs	Total Sig. Electrode Pairs	First Region Leads (Count)	First Region Leads (Percent)
**Net Granger Analysis**					
MFG - > ACC	0.99	397	260	157	0.6
MFG - > OFC	0.99	552	313	218	0.7
Amyg - > OFC	0.98	321	168	104	0.62
Amyg - > ACC	0.97	65	29	27	0.93
HC - > OFC	0.94	385	170	88	0.52
HC - > ACC	0.71	90	40	25	0.62
Amyg - > MFG	0.63	95	55	25	0.45
HC - > MFG	0.61	179	63	29	0.46
OFC - > ACC	0.41	396	265	132	0.5
HC - > Amyg	0.08	336	209	107	0.51
**Cross Correlation Analysis**					
MFG - > ACC	0.97	397	334	189	0.57
MFG - > OFC	0.96	552	464	281	0.61
Amyg - > OFC	0.96	321	259	148	0.57
Amyg - > ACC	0.96	65	50	33	0.66
ACC - > HC	0.94	90	58	36	0.62
Amyg - > MFG	0.82	95	70	41	0.59
Amyg - > HC	0.7	336	305	128	0.42
HC - > OFC	0.46	385	273	124	0.45
ACC - > OFC	0.2	396	324	159	0.49
HC - > MFG	0.17	179	105	35	0.33

Supp. Table 7. Results for both the net Granger and cross-correlation analysis for each region pair. P+ represents the proportion of posterior samples where the effect is greater than zero, and we consider P+ > 0.95 as strong evidence for directionality. For each region pair, we also include the total number of region pairs in the dataset, the total number of pairs that showed significant directionality, the number of pairs where the first region led the second region, and the percentage of pairs where the first region led the second region out of the total number of pairs with significant directionality.

Supplemental Figure 7. Estimated effect of time on theta coherence across different thresholds for elevated theta coherence

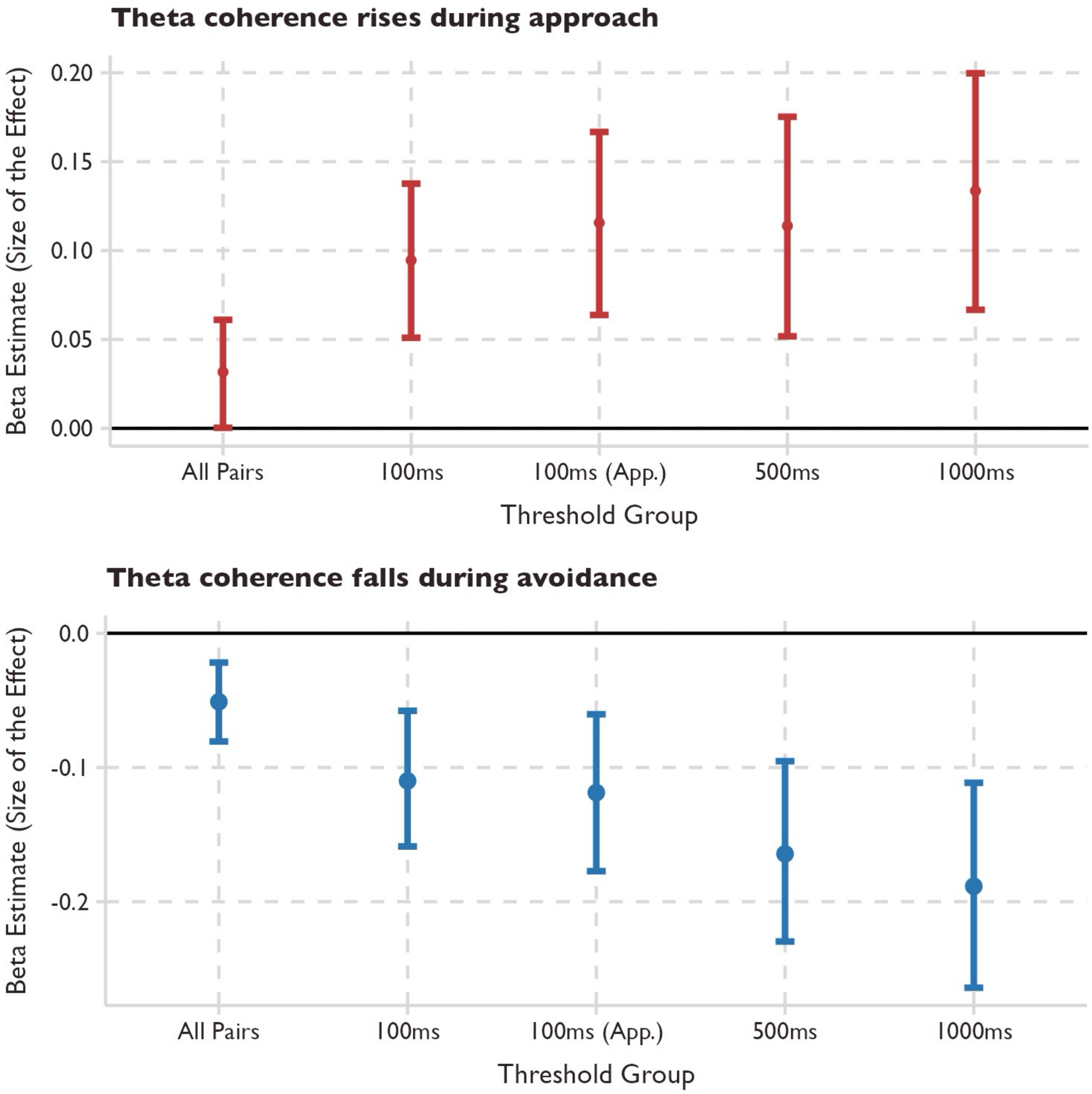

Supp. Figure 7: More stringent thresholds result in stronger estimated effects of time on theta coherence in both the approach (top) and avoidance (bottom) periods. The Y-axis is the beta estimate of the effect of time on theta coherence. The X-axis is the threshold group: 'All Pairs' shows the estimated effect without thresholding, '100ms' shows the estimated effect with a 100ms threshold (corresponding to the original result), '100ms (App.)' shows the estimated effect when thresholding by 100ms in the approach window only, and '500ms' and '1000ms' show the estimated effect when thresholding by 500ms and1000ms, respectively. Error bars represent the 95% confidence intervals around the effect.

Supplemental Table 8. P+s values for the Net Granger analysis across different theta coherence thresholds

Probability Positive Values by Threshold Group				
**Region Pair**	**100ms (Original)**	**100ms (App. Only)**	**500ms**	**1000ms**
MFG -> ACC	0.994	0.993	0.991	0.988
MFG -> OFC	0.993	0.995	0.994	0.994
Amyg -> OFC	0.975	0.968	0.974	0.974
Amyg -> ACC	0.972	0.974	0.947	0.943
HC -> OFC	0.937	0.955	0.927	0.896
HC -> ACC	0.706	0.722	0.55	0.548
Amyg -> MFG	0.628	0.646	0.688	0.776
HC -> MFG	0.609	0.538	0.666	0.689
OFC -> ACC	0.412	0.321	0.341	0.323
HC -> Amyg	0.079	0.072	0.076	0.076

Supp. Table 8: Probability Positive values, which describe the posterior distribution, for models estimating the directionality between region pairs, based on the Granger Causality analysis. The threshold group is defined in the following manner: '100ms' includes electrode pairs with 100ms of elevated theta coherence (corresponding to the original result), '100ms (App.)' includes electrode pairs with 100ms of elevated coherence in the approach window only, and '500ms' and '1000ms' include pairs with 500ms and1000ms of elevated theta coherence, respectively. Grey shading indicates the Probability Positive values were greater than 0.95 in both the original thresholding and in the cross-correlation analysis.

Supplemental Figure 8. Estimated correlation between of approach time and theta and HFA synchrony across different thresholds for theta coherence

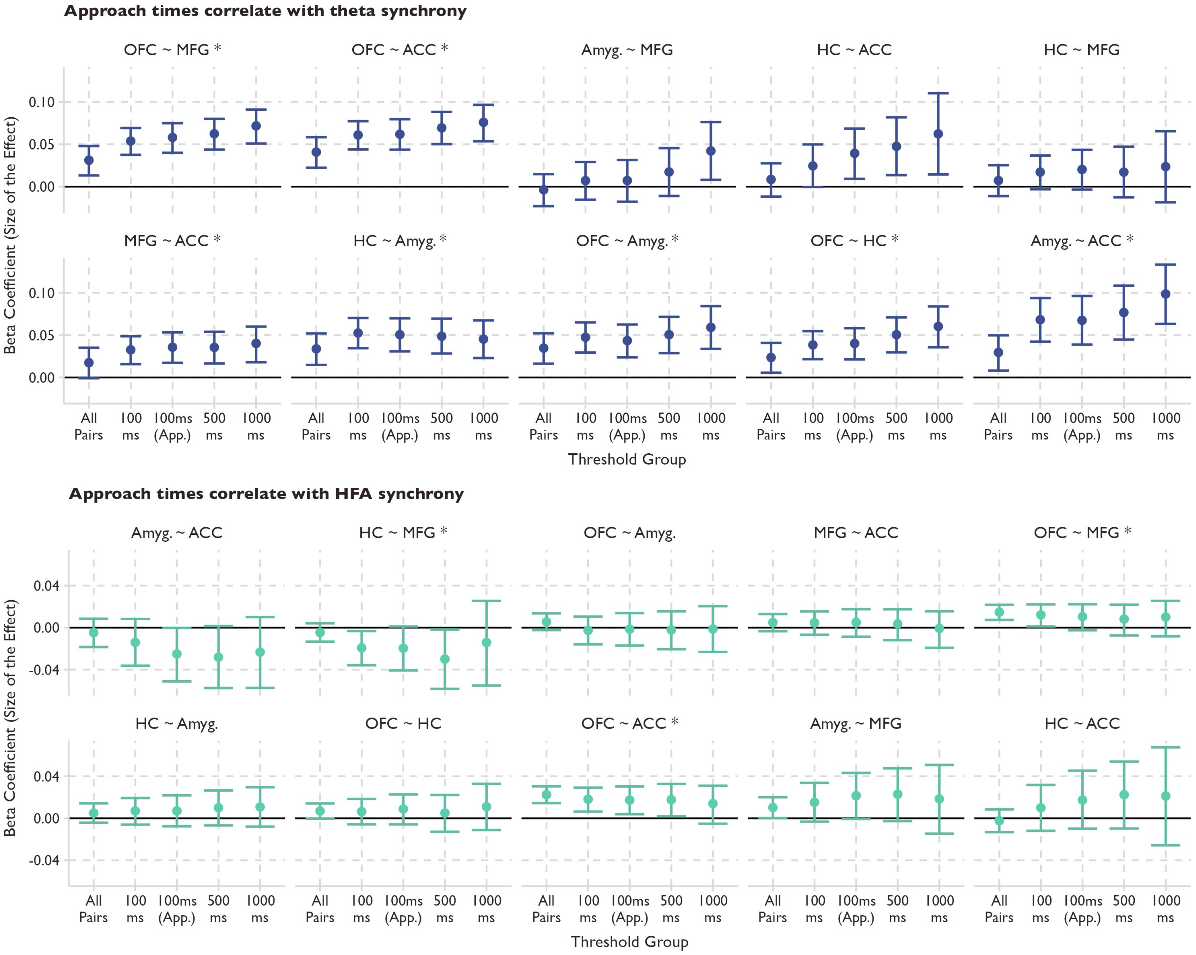

***Supp. Figure 8**:* Effect of different thresholds on estimated correlation between approach times and theta synchrony (top) and HFA synchrony (bottom). The Y-axis is the beta estimate of the correlation between of approach time and synchrony. The X-axis is the threshold group: 'All Pairs' shows the estimated effect without thresholding, '100ms' shows the estimated effect with a 100ms threshold (corresponding to the original result), '100ms (App.)' shows the estimated effect when thresholding by 100ms in the approach window only, and '500ms' and '1000ms' show the estimated effect when thresholding by 500ms and1000ms, respectively. Error bars represent the 95% confidence intervals around the effect. Stars indicate if the region pair was significant in the original analysis.

Supp Table 9. Posterior predictive checks for models estimating the effect of approach time on HFA & Theta synchrony

Posterior Predictive Check: Synchrony ~ Approach Time				
Statistic	Observed Value	Posterior Predictive Mean	Credible Interval	Posterior Predictive p value
**Theta**				
Mean	0.01	0.01	[0.007, 0.017]	0.45
Standard Deviation	1.00	1.00	[1.002, 1.009]	0.45
**HFA**				
Mean	0.02	0.02	[0.011, 0.02]	0.51
Standard Deviation	0.99	0.99	[0.982, 0.988]	0.51

Supp. Table 9. Posterior predictive checks on the mean and standard deviation for the models using approach time to predict theta and HFA synchrony. The observed value is the observed mean or standard deviation of the data. The mean, 95% confidence intervals, and posterior predictive values (which should be near .5) of the posterior distribution of theta coherence are shown. In all cases, the observed data match the fit posterior distribution.

Supplemental Figure 9. Clinical mood symptoms across online and intracranial cohorts

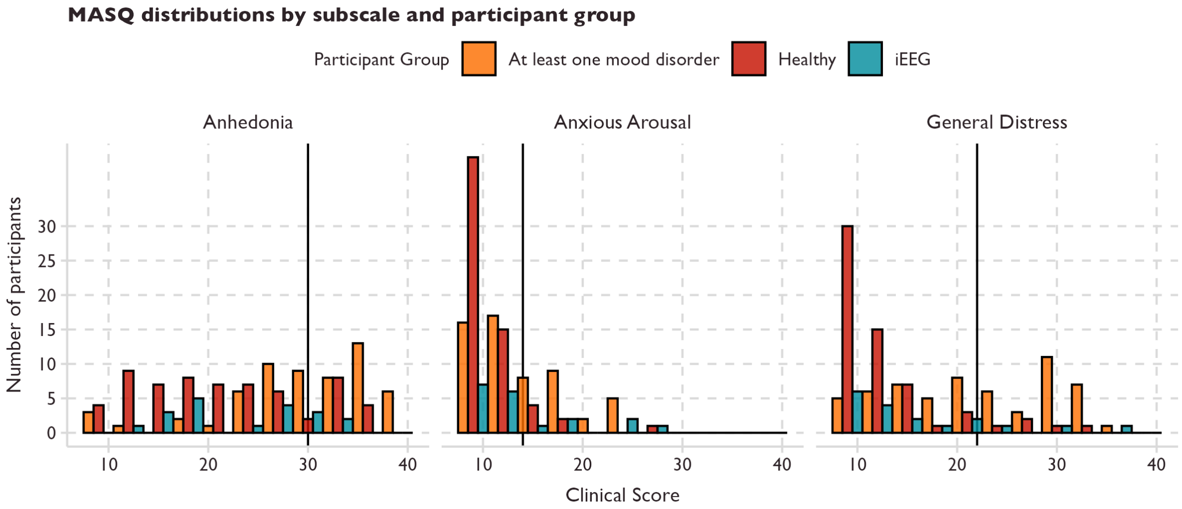

Supp. Figure 9: Clinical symptom histograms for each subscale of the MASQ across three participant groups. The Y-axis is the number of participants and X-axis is the subscale score. The online participants are broken into two groups, those who reported having at least one mood disorder (n=59), and those who did not (n= 63; marked 'Healthy' in the plot). The horizontal line denotes the average subscale score for the online participants who reported at least one mood disorder. Only 4/20 of the iEEG participants reported more symptoms than then the average symptoms in at least two subscales in the cohort with at least one mood disorder, and the distribution of symptoms in the iEEG sample is qualitatively similar to the healthy cohort.

Supplemental Figure 10. Number of turns per trial for subset of online participants

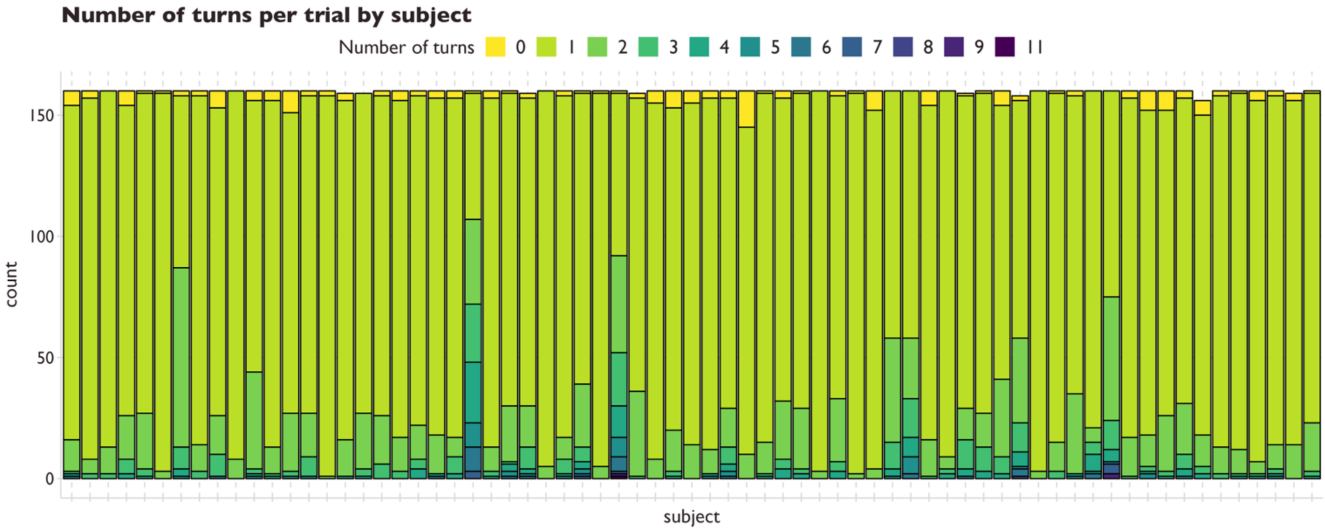

Supp. Figure 10*:* Distribution of the number of turns for a subsample of online participants. The Y-axis is the trial count, and the X-axis is a subsample of participants. The stacked bar chart shows the number of trials in which there were 0 turns (participant ran into the Ghost), a single turn, 2 turns, etc. Darker colors correspond to more turns within a single trial. While most participants synchronized their movements with the Ghost on some trials, the majority of participants approached and then turned to avoid a single time.
