# Supplementary material for: Cortical-limbic circuit dynamics of approach-avoidance conflict in humans
Source: Nat Commun. 2026 Mar 12;17:3867. doi: 10.1038/s41467-026-70287-5 (PMC13125241; doi:10.1038/s41467-026-70287-5)
Supplement: Supplementary file 2 — Reporting Summary [file 41467_2026_70287_MOESM2_ESM.pdf]

nature portfolio

Corresponding author(s): Brooke R. Staveland

Last updated by author(s): Mar 28, 2025

Reporting Summary

Nature Portfolio wishes to improve the reproducibility of the work that we publish. This form provides structure for consistency and transparency in reporting. For further information on Nature Portfolio policies, see our Editorial Policies and the Editorial Policy Checklist.

Statistics

For all statistical analyses, confirm that the following items are present in the figure legend, table legend, main text, or Methods section.

n/a	Confirmed	
☐	☐	The exact sample size (*n*) for each experimental group/condition, given as a discrete number and unit of measurement
☐	☒	A statement on whether measurements were taken from distinct samples or whether the same sample was measured repeatedly
☐	☒	The statistical test(s) used AND whether they are one- or two-sided<br>*Only common tests should be described solely by name; describe more complex techniques in the Methods section.*
☐	☒	A description of all covariates tested
☐	☒	A description of any assumptions or corrections, such as tests of normality and adjustment for multiple comparisons
☐	☒	A full description of the statistical parameters including central tendency (e.g. means) or other basic estimates (e.g. regression coefficient) AND variation (e.g. standard deviation) or associated estimates of uncertainty (e.g. confidence intervals)
☒	☐	For null hypothesis testing, the test statistic (e.g. *F*, *t*, *r*) with confidence intervals, effect sizes, degrees of freedom and *P* value noted<br>*Give P values as exact values whenever suitable.*
☐	☒	For Bayesian analysis, information on the choice of priors and Markov chain Monte Carlo settings
☐	☒	For hierarchical and complex designs, identification of the appropriate level for tests and full reporting of outcomes
☒	☐	Estimates of effect sizes (e.g. Cohen's *d*, Pearson's *r*), indicating how they were calculated

Our web collection on statistics for biologists contains articles on many of the points above.

Software and code

Policy information about availability of computer code

Data collection	Experimental task paradigm was coded in JavaScript and ran in a Chrome browser. Electrophysiological data were recorded using BCI2000, an open-source softwfare, at each clinical site. Online behavioral sample was collected using the online recruitment platform Prolific.
Data analysis	MNE-Python was used for preprocessing the neural data.The BRMS package in R was the main statistical package used. Github repos with the analysis code for preprocessing can be found at https://doi.org/10.5281/zenodo.17727554 and analysis code for all statistical tests and figures can be found at https://doi.org/10.5281/zenodo.17727552

For manuscripts utilizing custom algorithms or software that are central to the research but not yet described in published literature, software must be made available to editors and reviewers. We strongly encourage code deposition in a community repository (e.g. GitHub). See the Nature Portfolio guidelines for submitting code & software for further information.

Data

Policy information about availability of data

All manuscripts must include a data availability statement. This statement should provide the following information, where applicable:

- Accession codes, unique identifiers, or web links for publicly available datasets
- A description of any restrictions on data availability
- For clinical datasets or third party data, please ensure that the statement adheres to our policy

The cleaned, minimally-processed, patient-level data generated in this study have been deposited in the Zenodo database under accession [TBD]. The data used to generate each figure in this study are provided in the Source Data file.

Research involving human participants, their data, or biological material

Policy information about studies with human participants or human data. See also policy information about sex, gender (identity/presentation), and sexual orientation and race, ethnicity and racism.

Reporting on sex and gender	Sex (n=20, 10 female for intracranial patients; n=191, 93 female, 3 did not report for Online behavioral participants) was self reported and not considered in the study design due to opportunistic sampling strategy. Sex- and gender-based analyses were not performed due to the lack of statistical power in the intracranial sample. A table with patient demographics, including sex, is included in the supplement.
Reporting on race, ethnicity, or other socially relevant groupings	N/A
Population characteristics	Twenty intracranial participants had a mean age of 27.25 (SD = 12 years), had normal IQ (>85), spoke fluent English, and were all undergoing neurosurgical treatment for epilepsy. 191 Online behavioral participants had a mean age of 35.96 (SD = 12 years), were recruited from the United States, and were balanced for sex. All Online participants had to pass a short quiz to ensure understanding of the task.
Recruitment	Participants in the iEEG study were recruited from all patients undergoing intracranial monitoring for clinical treatment of epilepsy during the duration of the study that met the following inclusion criteria of being over 18 years old and spoke fluent English, meaning these results may not generalize beyond these populations. Only participants with electrode coverage in at least two of the following regions were included due to prior anatomical hypotheses: amygdala, hippocampus, orbitofrontal cortex, anterior cingulate cortex, and middle frontal gyrus. Participants were informed about the possibility to participate in basic research before undergoing implantation of stereotactic EEG electrodes for the localization of seizure foci. Informed consent was obtained from those still interested in participating after implantation of the electrodes. We additionally collected 191 participants, balanced for from the online recruitment platform, Prolific, using the 'Representative sample' option, which balances the sample with regard to demographics like sex, based on census data from the US.
Ethics oversight	University of California, Berkeley, Loma Linda University Medical Center, Barnes-Jewish Hospital in St. Louis, and St.Lous Children's Hospital.

Note that full information on the approval of the study protocol must also be provided in the manuscript.

Field-specific reporting

Please select the one below that is the best fit for your research. If you are not sure, read the appropriate sections before making your selection.

☐ Life sciences ☒ Behavioural & social sciences ☐ Ecological, evolutionary & environmental sciences

For a reference copy of the document with all sections, see nature.com/documents/nr-reporting-summary-flat.pdf

Behavioural & social sciences study design

All studies must disclose on these points even when the disclosure is negative.

Study description	This study collected quantitative behavioral and intracranial electroencephalography (iEEG) data from 20 participants. Additional behavioral data collected from 191 participants via the online recruitment platform Prolific.
Research sample	See above.
Sampling strategy	Due to the rare nature of these data, a convenience sample was recruited from all potentially eligible patients being treated for epilepsy at the hospitals. Participants in the iEEG study were required to meet the inclusion criteria of being over 18 years old and speak fluent English. Same size is adequate due to the high signal-to-noise characteristics of iEEG data and the strong statistical power provided by Bayesian modeling of linear mixed effects models.
Data collection	Electrophysiological data were recorded using BCI2000, an open-source software, at each clinical site (Schalk et al., 2004). This

	system synchronized the task with LFPs, eye tracking, and behavior (Pac-Man movement) in a single data stream which supports easy data pooling across sites and data sharing with the community (Milsap et al., 2019). The sampling rate at Barnes-Jewish Hospital was 2000Hz, while at Loma Linda the sampling rate was 512Hz.
Timing	Intracranial data were collected from November 2021 until May 2024. An initial pilot sample of 69 participants were recruited from Prolific in the Fall of 2021. And additional 122 participants were recruited Fall 2023.
Data exclusions	Raw iEEG traces were manually inspected by neurologist for epileptic spiking and spread, as well as artifacts (e.g., machine noise, signal drift, amplifier saturation, etc). Data in regions or epochs with epileptiform or artifactual activity were excluded from further analyses. Finally, trials were rejected for task interruptions (task was paused) or if no rewards were collected.
Non-participation	No participants dropped out or declined participation.
Randomization	Participants were not allocated into groups due to the within-subjects experimental design in which all participants performed all conditions.

Reporting for specific materials, systems and methods

We require information from authors about some types of materials, experimental systems and methods used in many studies. Here, indicate whether each material, system or method listed is relevant to your study. If you are not sure if a list item applies to your research, read the appropriate section before selecting a response.

Materials & experimental systems

n/a	Involved in the study
☒	☐ Antibodies
☒	☐ Eukaryotic cell lines
☒	☐ Palaeontology and archaeology
☒	☐ Animals and other organisms
☒	☐ Clinical data
☒	☐ Dual use research of concern
☒	☐ Plants

Methods

n/a	Involved in the study
☒	☐ ChIP-seq
☒	☐ Flow cytometry
☒	☐ MRI-based neuroimaging

Plants

Seed stocks	*Report on the source of all seed stocks or other plant material used. If applicable, state the seed stock centre and catalogue number. If plant specimens were collected from the field, describe the collection location, date and sampling procedures.*
Novel plant genotypes	*Describe the methods by which all novel plant genotypes were produced. This includes those generated by transgenic approaches, gene editing, chemical/radiation-based mutagenesis and hybridization. For transgenic lines, describe the transformation method, the number of independent lines analyzed and the generation upon which experiments were performed. For gene-edited lines, describe the editor used, the endogenous sequence targeted for editing, the targeting guide RNA sequence (if applicable) and how the editor was applied.*
Authentication	*Describe any authentication procedures for each seed stock used or novel genotype generated. Describe any experiments used to assess the effect of a mutation and, where applicable, how potential secondary effects (e.g. second site T-DNA insertions, mosiacism, off-target gene editing) were examined.*
